# Catalytically Enhanced Hydrogen Sorption in Mg-MgH_2_ by Coupling Vanadium-Based Catalyst and Carbon Nanotubes

**DOI:** 10.3390/ma8063491

**Published:** 2015-06-12

**Authors:** Atikah Kadri, Yi Jia, Zhigang Chen, Xiangdong Yao

**Affiliations:** 1 Centre of Excellence for Functional Nanomaterials, the University of Queensland, St. Lucia, Brisbane, QLD 4067, Australia; E-Mails: atikahkadri@salam.uitm.edu.my (A.K.); y.jia@griffith.edu.au (Y.J.); z.chen1@uq.edu.au (Z.C.); 2 Faculty of Chemical Engineering, Universiti Teknologi Mara (UiTM), Shah Alam, Selangor 40000, Malaysia; 3 Queensland Micro- and Nanotechnology Centre (QMNC), Griffith University, Nathan, Brisbane, QLD 4111, Australia

**Keywords:** hydrogen storage, magnesium hydride, carbon nanotubes, vanadium complex catalyst

## Abstract

Mg (MgH_2_)-based composites, using carbon nanotubes (CNTs) and pre-synthesized vanadium-based complex (VCat) as the catalysts, were prepared by high-energy ball milling technique. The synergistic effect of coupling CNTs and VCat in MgH_2_ was observed for an ultra-fast absorption rate of 6.50 wt. % of hydrogen per minute and 6.50 wt. % of hydrogen release in 10 min at 200 °C and 300 °C, respectively. The temperature programmed desorption (TPD) results reveal that coupling VCat and CNTs reduces both peak and onset temperatures by more than 60 °C and 114 °C, respectively. In addition, the presence of both VCat and CNTs reduces the enthalpy and entropy of desorption of about 7 kJ/mol H_2_ and 11 J/mol H_2_·K, respectively, as compared to those of the commercial MgH_2_, which ascribe to the decrease of desorption temperature. From the study of the effect of CNTs milling time, it is shown that partially destroyed CNTs (shorter milling time) are better to enhance the hydrogen sorption performance.

## 1. Introduction

One of the challenges that need to be addressed for materializing zero-emission in mobile application is the mode of energy storage. Solid-state hydrogen storage is an attractive option as it offers a safe and efficient mode of storing hydrogen for on board applications. Hydrogen storage in metal hydride, particularly in magnesium hydride (MgH_2_), has been the focus of intensive research for decades. Possessing both high gravimetric (7.6 wt. % of hydrogen) and volumetric (110 kg/m^3^) densities with an excellent reversibility and low cost makes MgH_2_ an attractive storage candidate. However, the major drawbacks in the practical application of such material are its sluggish hydrogenation/dehydrogenation kinetics and high operational temperature. This is attributed to the fact that there is a very strong ionic bonding between magnesium and hydrogen. Thus bulk MgH_2_ requires a decomposition enthalpy of about 75 kJ/mol H_2_, which corresponds to a desorption temperature of above 300 °C at one bar [1].

A great deal of research efforts, as reported in the literature, has been directed toward improving MgH_2_ hydrogen storage properties. Strategies include reducing particle size and adding additives to serve as active sites. Among the additives that have been investigated are transition metals [2], oxides [3,4,5,6] and metal halides [7,8,9,10]. Zaluska and Zaluski [11] have introduced a complex catalyst that utilizes both hydride-forming metal and an electronegative element such as oxygen, nitrogen or carbon bridged with hydrogen, which can be described by the following structural formula: T-H—E, where T, H and E represent metal, hydrogen and electronegative element, respectively. The hydrogen in the complex catalyst is believed to be ambivalent in the presence of a strongly electronegative element and thus has a tendency to adopt the electropositive character and form a protonic bond with the electronegative element. During absorption, the function of this complex catalyst is to stimulate and enable ionization of the dissociating hydrogen, while in the desorption process hydrogen has to be attracted, thus leading to the recombination into hydrogen molecules [11].

The unique characteristics of combining carbon materials in a hydrogen storage material have drawn interest amongst research groups and this topic was recently reviewed in detail [12,13]. Mitlin *et al.* [14] have studied the performance of 1 h co-milling MgH_2_ with unpurified CNTs containing 10% metallic nanoparticles. The researchers found that at longer co-milling times, the CNTs were completely destroyed and the enhancement was significantly lost. The positive traits of single-walled carbon nanotubes (CNTs) in MgH_2_ system are reported not to alter the microstructure of MgH_2_, instead it acts as surface dispersant and/or ‘hydrogen pumps’ in the composites [15]. An investigation of combining cobalt and CNTs discovered that the main role of cobalt is to promote the hydrogen dissociation/combination on its surface, whereas the presence of CNTs increases the hydrogen storage capacity and facilitates hydrogen diffusion in the bulk MgH_2_ [16]. In our previous work [17], we reported that the synergistic effect of combining individual metal nanoparticles (Fe and Ti) and CNTs on the kinetics as well as in hydrogen storage capacity. In addition to the physical contributions of the CNTs in facilitating mechanical milling, it was also suggested that the CNTs provide fast diffusion surface/channels for hydrogen to reach to Mg or MgH_2_.

A common method for reducing particle size in Mg-based materials is to employ a mechanical milling technique. Theoretical studies have reported that using nanoparticles instead of bulk or coarse particles of hydrides can alter the thermodynamics of hydrogen uptake and release [18,19,20]. In addition to reducing the particle and grain size, ball milling may also create a defect and thus facilitate nucleation during the absorption and desorption process. However, most reported investigations require long milling times ranging between 10 to 80 h [16,21,22] in preparation of a storage material with reasonable sorption performance. This is obviously not economical when considering mass production. There are shorter milling times between 1 and 2 h reported in the literature, however, the sorption performances are unattractive even at 300 °C [14,23,24] except the recent works of adding nanoscale catalysts [25].

In this work, the hydrogen sorption of magnesium hydride composites containing both vanadium-based complex catalyst and CNTs were investigated. An attempt to reduce the milling time while maintaining excellent sorption performance is also one of the aims of this study. The vanadium-based complex catalysts (denoted as VCat) are prepared from the hydride metal of vanadium and copper oxide as the source of the electronegative element (oxygen). The choice of vanadium is due to its high efficiency for catalytic hydrogen storage [25,26,27], while CuO may also provide the synergistic catalytic effect of bi-metals as reported bi-metals have much more catalytic effect on de/hydrogenation than its individuals [17,22]. The *in situ* formed metallic particles of VCat are all at nanoscale thus possibly reduce the sample milling time while maintaining high hydrogen storage performance. The function of CNTs was identified by investigating the effect of CNTs milling time on hydrogen storage of the composites. 

## 2. Experimental 

### 2.1. Synthesis of VCat

VCat was prepared from the hydride metal of vanadium hydride (VH_2_) and copper oxide as the source of electronegative element (oxygen), while water and methanol solution acted to control the contribution of oxygen through a self-adjusting mechanism [28]. Four hundred fifty milligrams of vanadium hydride (VH_2_, Sigma Aldrich, St. Louis, MO, USA) and 350 mg of Copper (II) oxide nanopowders (CuO, Sigma Aldrich, St. Louis, MO, USA), together with stainless steel balls of a ball-to-powder ratio of about 20:1 on weight basis, were loaded in stainless steel vial. Then, 0.5 mL of methanol solution was loaded into the vial. Subsequently, the vial was mounted in a high-energy ball mill (SPEX 8000 Mixer/Mill, SPEX SamplePrep, Metuchen, NJ, USA) and ball milled for 9 h. The detailed procedures can be found in ref [29].

### 2.2. Synthesis of MgH_2_ Storage Composites

Magnesium hydride (MgH_2_, hydrogen storage grade, Sigma Aldrich), prepared catalyst (VCat) and/or CNTs (prepared and purified in our lab) were used to prepare the MgH_2_-based composites. Ball milling of MgH_2_ (denoted as M), MgH_2_ + VCat (denoted as MV5), MgH_2_ + CNTs (denoted as Mcnt5) and MgH_2_ + VCat + CNTs (denoted as MVcnt5) was performed in a high energy SPEX 8000 vibration ball mill under an argon atmosphere. To reduce the milling time, a shorter milling time of 5 h was selected (compared to long milling times reported in the literature of a similar system) [16,22]. The amount of additives introduced to MgH_2_ was 5 wt. % each and the ball to powder ratio was kept at 40:1 in all samples. Another sample was prepared with shorter CNTs milling time, in which CNTs were introduced to MgH_2_ + VCat after a 4.5 h milling period to give only 0.5 h of CNTs milling time; this sample is denoted as MV(4.5)cnt(0.5). All sample handling for both VCat and MgH_2_ storage material was performed in an Argon-filled glove box (MBraun), in which the water and oxygen level were kept below 1 ppm to minimize the possible oxidation of samples.

### 2.3. Characterization

The microstructures of the samples were characterized in an air-sensitive sample holder by X-ray diffraction (XRD, Rigaku MiniFlex, Rigaku, Tokyo, Japan) with Co Kα radiation at a scanning rate of 0.1°/min in the 2θ range from 20 to 90°. The morphology and composition of the samples were further analyzed by employing a transmission electron microscope (TEM, FEI F20, FEI, Hillsboro, OR, USA) equipped with an energy dispersive X-ray spectroscopy (EDS, Oxford instruments, Oxfordshire, UK) analysis unit (AMETEK). The sample for TEM measurement was dispersed in heptane by ultrasonication and then deposited onto a holey carbon film on a copper grid. The preparation of XRD and TEM samples were performed in an Ar-filled glovebox (MBraun), in which the water/oxygen levels were below 1 ppm. Raman analysis was carried out using Nicolet Almega™ Visible Raman Spectrometer (Thermo Scientific, Waltham, MA, USA) equipped with a charge-coupled device (CCD) detector having a red laser with an excitation wavelength of 633 nm.

All hydrogenation measurements of the milled powders were evaluated using an automated Sieverts apparatus (Suzuki Shokan PCT H_2_ Absorption Rig). Before measurements, the system was degassed for 2 h with the sample cell being heated to 350 °C. Absorption and desorption test were performed at various temperatures under an initial pressure of 2.0 MPa and 1 KPa, respectively. Non-isothermal hydrogen desorption of the samples was measured by temperature programmed control desorption with mass spectrometry (TPD-MS) performed by a Brooks 5850E mass flow controller, which was attached with a quartz reactor in a tube furnace. Argon was used as the carrier gas and all samples were subjected to a 5 °C/min heating rate with 50 mL/min flowing rate during the measurements. 

## 3. Result and Discussion

### 3.1. Characterization of Vanadium-Based Complex Catalyst

**Figure 1:**
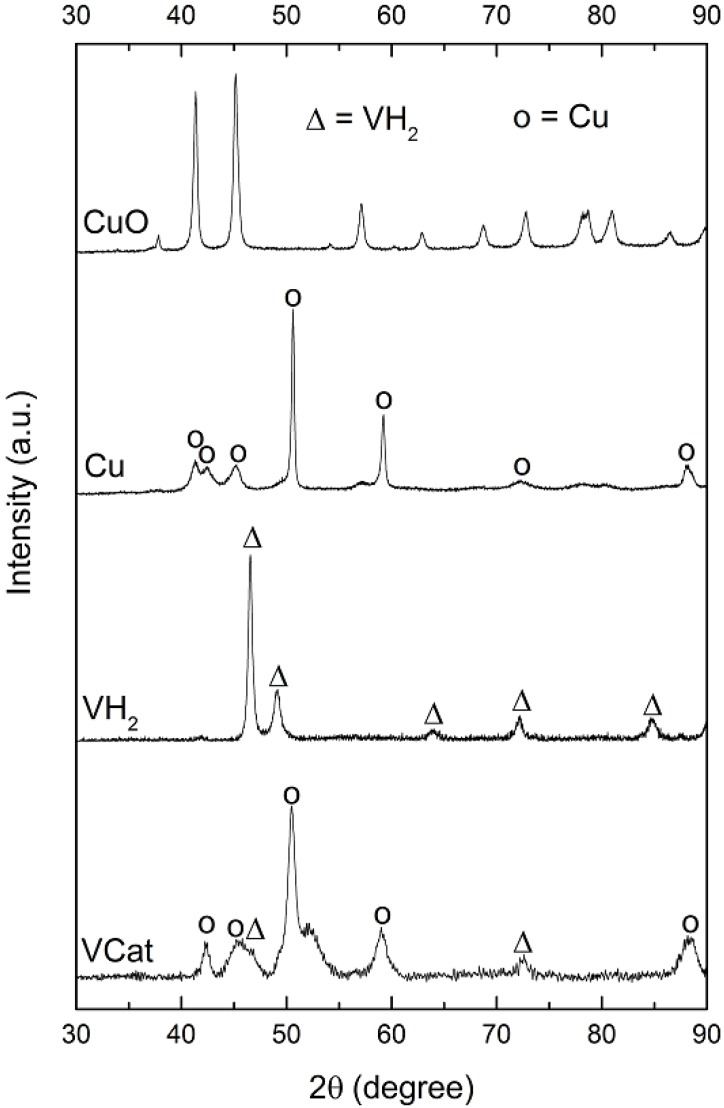
XRD profiles of VCat, VH_2_, Cu and CuO.

Figure 1 shows the XRD patterns of the prepared catalyst (VCat), the initial materials and the possible product of the catalyst synthesis for comparison. The prepared catalyst shows combination peaks of VH_2_ (2θ = 46.3° and 72.5°) and peaks of copper at 2θ of 42.4°, 45.6°, 50.5°, 59.1° and 88.4°. It is clearly shown that there is no observation of a CuO peak in the prepared catalyst, indicating that CuO has completely reduced to Cu during the ball milling. The most possible reducing agent is the hydrogen in methanol solution due to no appearance of V metal peak (the hydrogen is not from VH_2_ because it is not decomposed). Following the suggested structural formula made earlier, the synthesized catalyst can be represented by V-H—O, where vanadium and oxygen are bridged by hydrogen, thus such a coordinating structure allows to locally manipulate the charge transfer during the hydrogenation and dehydrogenation process.

### 3.2. Characterization of MgH_2_-Based Composites

**Figure 2:**
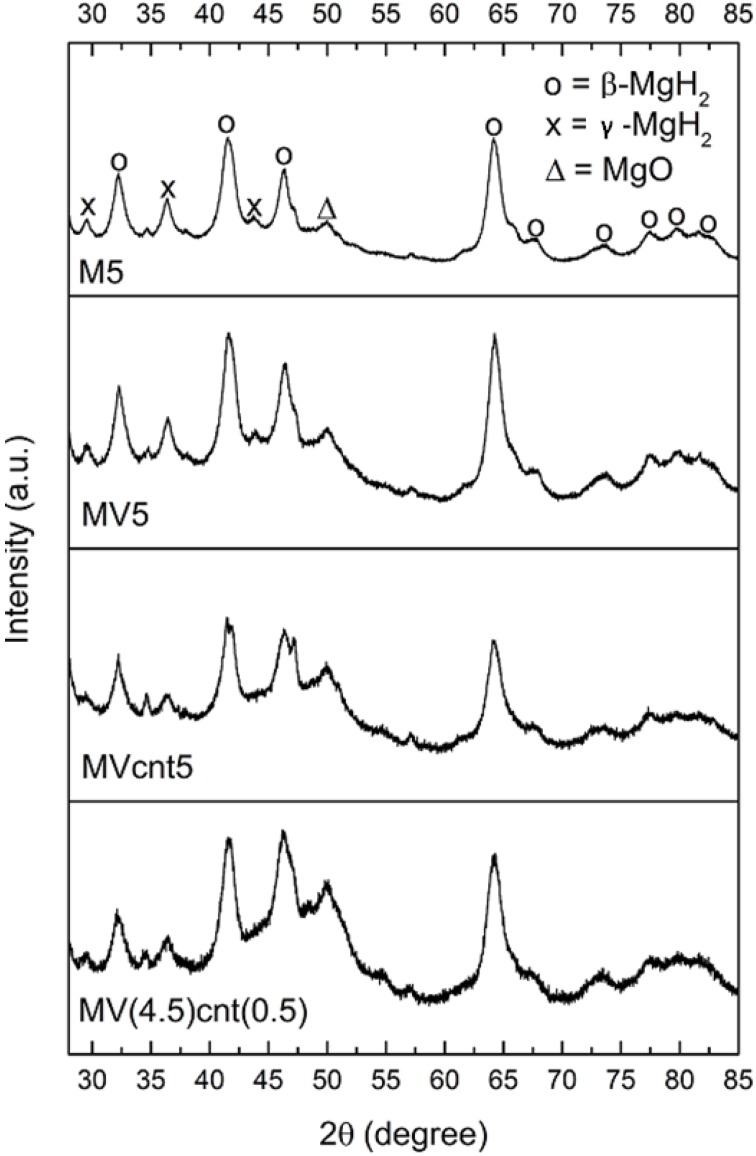
XRD profiles of MgH_2_ and MgH_2_-based composites milled for 5 h.

**Figure 3:**
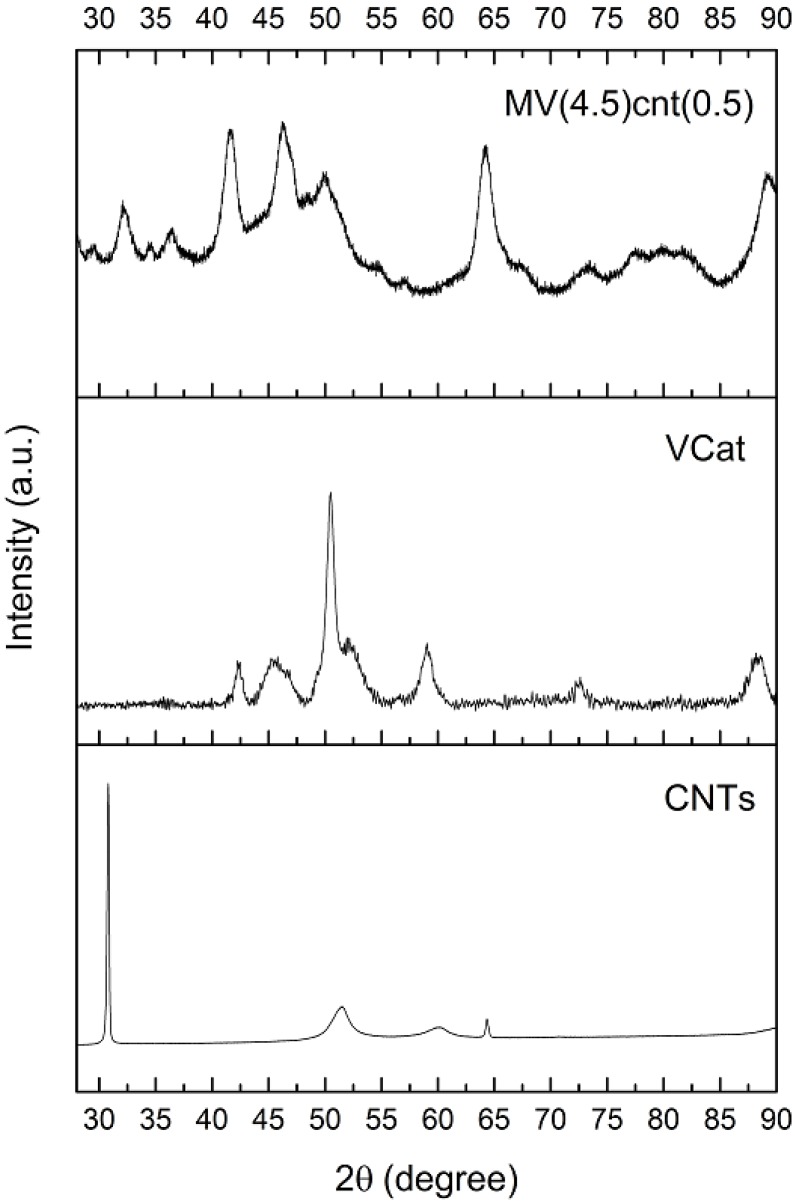
XRD profiles of CNTs, VCat and as milled MV(4.5)cnt(0.5).

The XRD patterns of the MgH_2_ and magnesium hydride composites, MV5, Mcnt5, MVcnt5 and MV(4.5)cnt(0.5), are shown in Figure 2. All patterns show similar peaks with the main peaks match β- MgH_2_ (JCPDS-12-0697) and other peaks correspond to γ-MgH_2_ (JCPDS-35-1184) and MgO (JCPDS-65-0476). The presence of the orthorhombic γ-phase is due to the alteration in the microstructure caused by the high energy ball milling [30]. It is noteworthy that there is no peak that indicates the formation of a new phase as a result of the reaction between MgH_2_ and VCat being observed. The considerably broad diffraction peaks of the samples is a result of a reduction in particle size as well as an increase in defects and mechanical strains created within the lattice during ball milling [31]. The crystallite sizes of β-MgH_2_ were estimated by a modified Scherrer equation [17] according to the diffraction pattern, the results of which are tabulated in Table 1. It shows that the grain sizes of β-MgH_2_ are close to one and another except for M5 having a grain size of 11.6 nm instead. Figure 3 illustrates the XRD patterns of CNTs, VCat and as-milled MV(4.5)cnt(0.5). There is no peak of CNTs as well as VCat can be observed in the as-milled MV(4.5)cnt(0.5), indicating both CNTs and VCat are well incorporated or dispersed in the prepared MgH_2_-based composites. It was reported that the addition of single walled carbon nanotubes containing metallic nanoparticles does not appreciably affects the bulk MgH_2_ microstructure, however it does act as a ‘lubricant’ to avoid particles from mechanically aggregate together [14].

**Table 1:** Grain size of β-MgH_2_ in various samples.

Sample	β-MgH_2_ Grain Size (nm)
M5	11.6
MV5	10.0
MV(4.5)cnt(0.5)	9.3
MVcnt5	9.5

**Figure 4:**
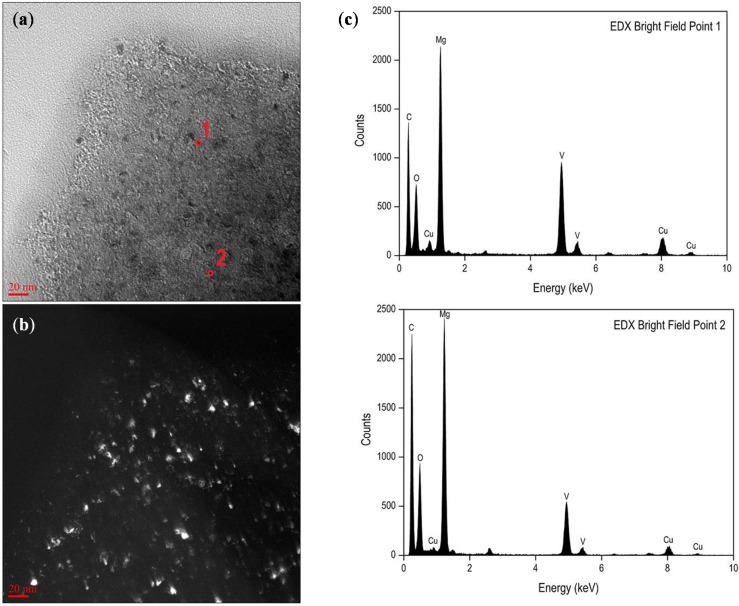
TEM images: (**a**) bright field; and (**b**) dark field of as milled MV(4.5)cnt(0.5); (**c**) EDX element analysis corresponding to spots on (**a**).

TEM analysis was conducted to further investigate the morphology of the synthesized MgH_2_-based composites. The TEM bright field and dark field images of the as-milled MV(4.5)cnt(0.5) are shown in Figure 4a,b, respectively. The grain size of MgH_2_ is observed to be in the range of less than 10 nm and this agrees well with the analysis made by XRD. The EDX element analyses were performed in the same sample to observe the dispersion of VCat in the synthesized composites and are shown in Figure 4c with the corresponding selected spots as shown in Figure 4a. It is clearly shown that VCat is well distributed in the synthesized composites and thus in close contact with MgH_2_ crystallites. VCat may act as nucleation and growth centers of the magnesium hydride phase as proposed by Schimmel *et al.* [32]. Therefore, based on the structural and morphological investigation, it is speculated that MV(4.5)cnt(0.5) will offer an improved hydrogen sorption properties as a result of the homogeneous dispersion of the catalysts within the smaller crystallite size of MgH_2_.

### 3.3. Tracing the Degree of Damage of CNTs in MgH_2_-Based Composites

**Figure 5:**
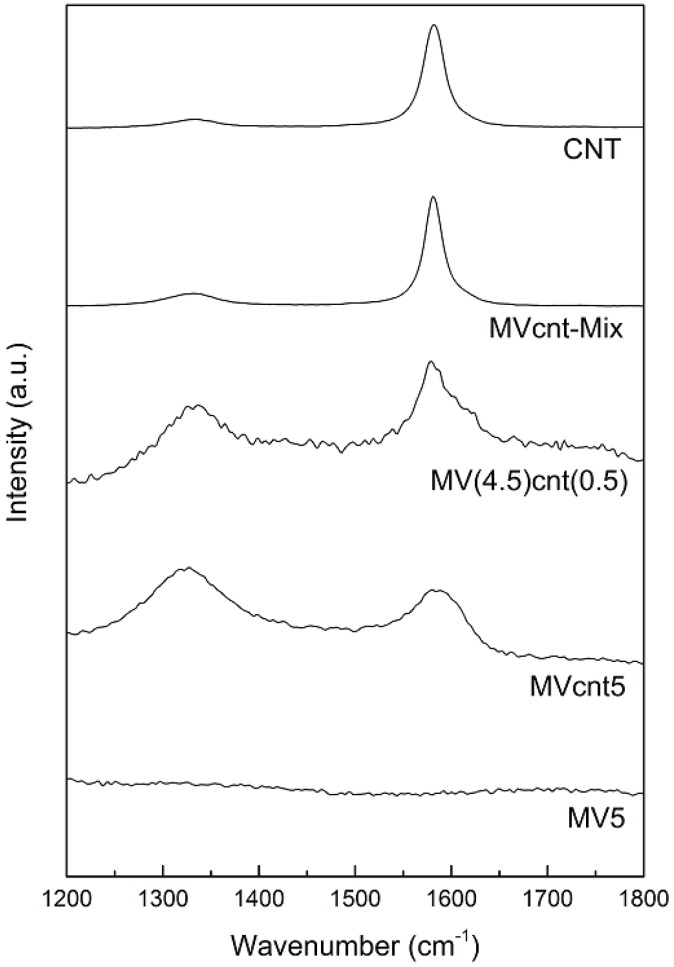
Raman spectra of MgH_2_-based composites and CNTs.

The Raman spectroscopy is an alternative method for analyzing the damage degree of CNTs. There are two particularly important peaks in the range of 1300 to 1700 cm^−1^ of the spectrum, which provides the fingerprint of carbon species. The peak located at around 1590 cm^−1^ corresponds to the tangential C-C stretching mode known as G band, which is characteristic of the graphitic structure in CNTs. The other peak around 1350 cm^−1^ designated as D band is assigned to the residual ill-organized graphite which is caused by impurities and defects on CNTs [33]. The relative intensity ratio of the D to the G bands, I_D_/I_G_ can provide information of CNTs structural defects, and, as the ratio increases it indicates the increase of the CNTs defect and the decrease of the degree of graphitization of carbon in the composites [21,34,35]. Figure 5 presents the Raman spectra of magnesium hydride composites and CNTs. All samples contained in the CNTs showed both G and D bands. The intensity of G band and D band in MVcnt-Mix (just physically mixed MgH_2_, Vcat and CNTs) are similar to the CNTs, which show that CNTs in the composite did not experience a structural defect. As the composite in MVcnt-Mix was prepared by agate mortar there, no high impact mixing occurred to allow the breakdown of CNTs to form amorphous carbon, thus most CNTs remained their original structure in the composite. However, samples prepared by the high-energy ball milling technique showed an increase of D band intensity. The high-energy milling caused some of the CNTs in the composites to breakdown to amorphous carbon and leave defective CNTs behind. Limiting the CNTs milling time may control the degree of tubular structure of CNTs being damaged or destroyed. Raman result shows the I_D_/I_G_ for MV(4.5)cnt(0.5) is less than MVcnt5 with a ratio of 0.95 and 1.11, respectively. It is clearly shown that by milling the CNTs for 5 h destroys more CNTs structure and at the same time retains less damaged CNTs structure compared to 30 min milling time. Several reports have published on the effect of ball milling on CNTs, which also demonstrate the planar structure is severely damaged during long period of milling [36,37,38]. Nevertheless, the tubular structure may be retained by applying a shorter milling time [14,36].

### 3.4. Hydrogen Storage Properties

**Figure 6:**
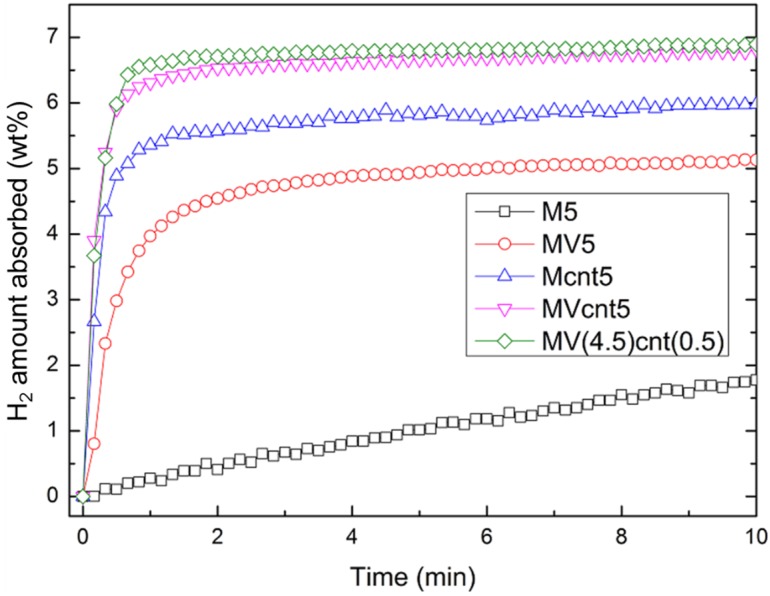
Hydrogen absorption of the different MgH_2_-based composites at 200 °C with an initial pressure of 2.0 MPa.

**Figure 7:**
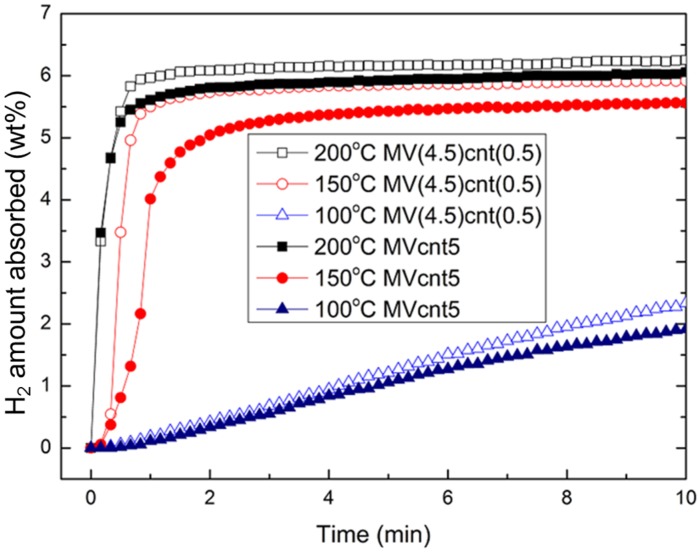
Hydrogen absorption of MVcnt5 and MV(4.5)cnt(0.5) at 200, 150 and 100 °C with an initial pressure of 2.0 MPa, respectively.

The hydrogen absorption rate is one criterion that indicates the performance of a hydrogen storage material. The hydrogen absorption rate of the synthesized magnesium-based composites at 200 °C is shown in Figure 6. M5 exhibits the least hydrogen absorption rate, while magnesium-based composites with coupling of both VCat and CNTs shows a higher absorption rate compared to their individual counterpart. Both MVcnt5 and MV(4.5)cnt(0.5) absorbed about 6.80 wt. % of hydrogen in 10 min compared to 5.50 wt. % and 4.60 wt. % of hydrogen for Mcnt5 and MV5, respectively. This result suggested that there is a synergistic effect of coupling both additives, which contributes to the superior absorption performance. Focusing on the impact of CNTs milling time, the absorption of composites with 0.5 h CNTs milling time showed better kinetics with 6.50 wt. % of hydrogen absorbed in 1 min compared to 6.20 wt. % for 5 h CNTs milling time. The effect of shorter CNTs milling time is more obvious at a lower absorption temperature of 150 °C. Figure 7 shows the hydrogen absorption of both composites of MVcnt5 and MV(4.5)cnt(0.5) at 200, 150 and 100 °C, respectively. It is noteworthy that the samples with shorter CNTs milling times, such as MV(4.5)cnt(0.5), show better hydrogen absorption kinetics, particularly a superior absorption rate is observed in MV(4.5)cnt(0.5) at 150 °C even close to the absorption performance of MVcnt5 at 200 °C. As observed in previous section, 30 min CNTs milling time retains more defective tubular CNTs structure in the composite compared to 5 h. It was reported that the CNTs defects form a low-energy sites, which easily interacts with molecules, radicals or metals [39,40,41]. The metallic catalysts act as hydrogen molecules splitting agents, which contact intimately with the defective tubular CNTs structure contributing further to the improved absorption rate by providing hydrogen diffusion channels to accelerate the absorption process. A similar observation was reported by Yao *et al.* with a proposed hydrogenation mechanism [17]. Additionally, the effect of coupling VCat and CNTs in this work show superior absorption performance even at as low as 150 °C.

**Figure 8:**
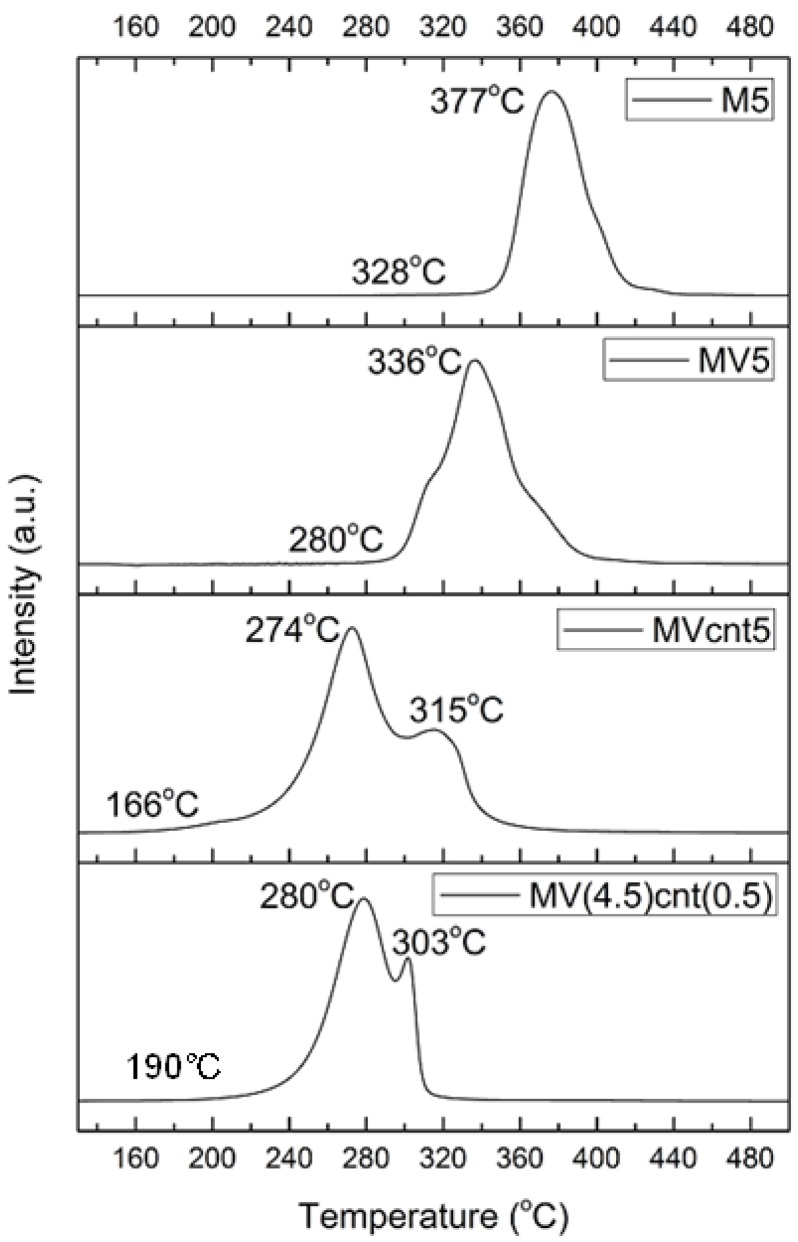
TPD-MS profiles of M5, MV5, MVcnt5 and MV(4.5)cnt(0.5).

Non-isothermal hydrogen desorption was investigated by temperature programmed control desorption with mass spectrometry (TPD-MS) using argon as the carrier gas. Figure 8 illustrates the TPD-MS profile of M5, MV5, MVcnt5 and MV(4.5)cnt(0.5) in the temperature ranges from 150 °C to 500 °C, with M5 exhibiting the highest desorption peak temperature of 377 °C. Milling MgH_2_ with VCat alone did not make much change to the peak of desorption temperature with only a drop of about 30 °C from peak temperature of M5. However, combining VCat and CNTs reduces the peak temperatures for more than 100 °C as shown in sample MVcnt5. With respect to the onset temperature, MVcnt5 shows a significant decrease of 162 °C and 114 °C compared to M5 and MV5, respectively, suggesting that coupling of VCat and CNTs enables the composites start to release hydrogen as low as ~166 °C. The effect of CNTs milling time inhibited further reduction in the peak and onset temperatures of desorption, however, there is a clear difference in the shape of both the TPD-MS peaks. MV(4.5)cnt(0.5) shows a sharp peak compared to MVcnt5, implying that the kinetics of hydrogen desorption improves more significantly with a shorter CNTs milling time.

**Figure 9:**
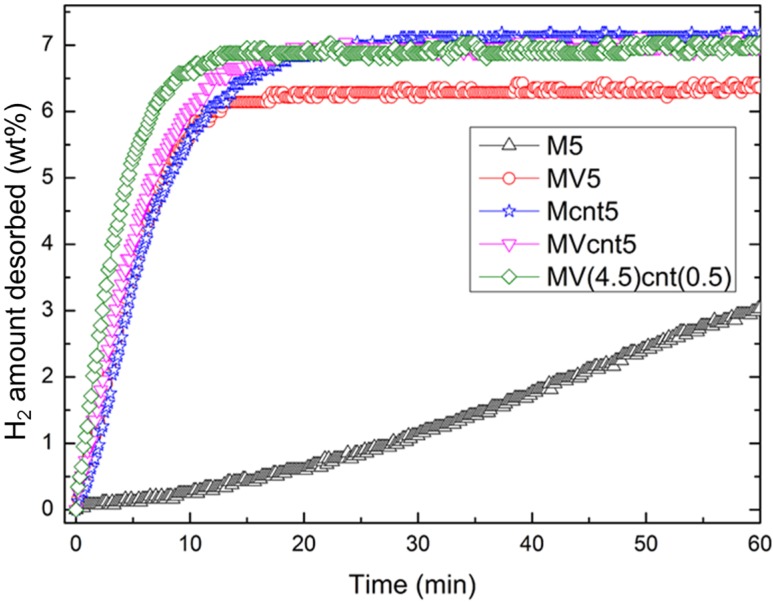
Hydrogen desorption of M5, MV5, Mcnt5, MVcnt5 and MV(4.5)cnt(0.5) at 300 °C with an initial pressure of 1 KPa, respectively.

**Figure 10:**
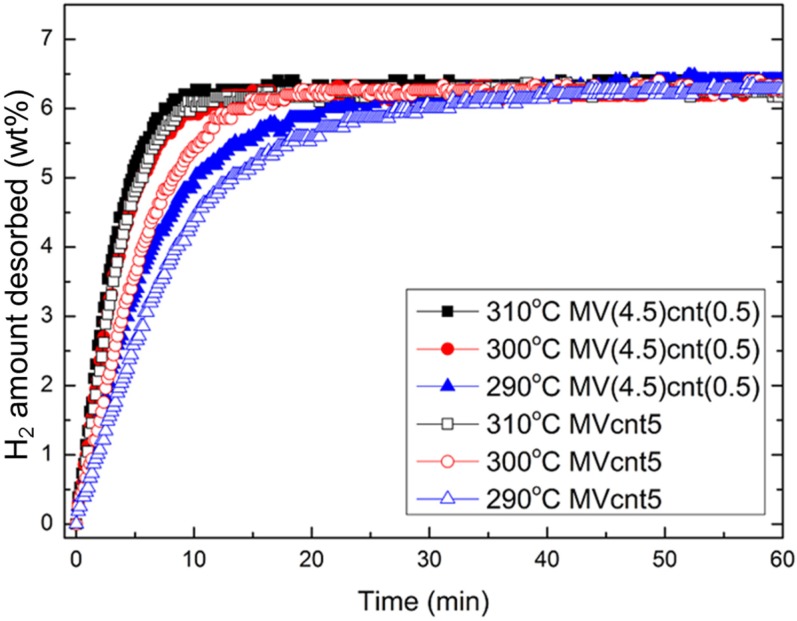
Hydrogen desorption of MVcnt5 and MV(4.5)cnt(0.5) at 310, 300 and 290 °C with an initial pressure of 1 KPa, respectively.

**Figure 11:**
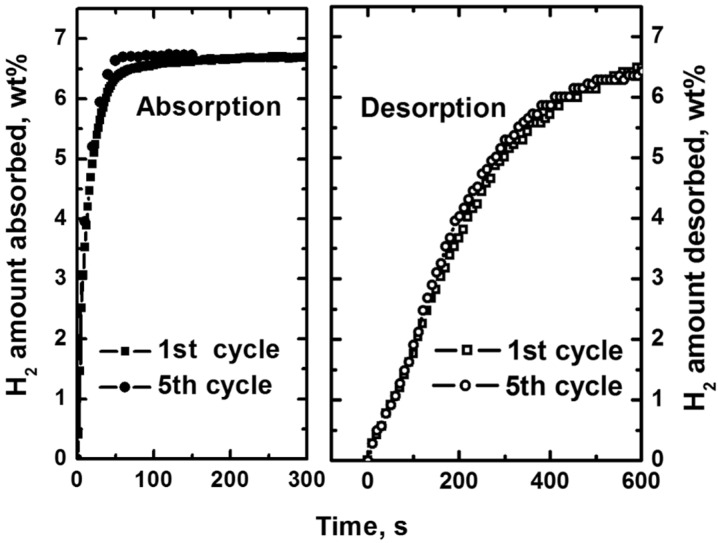
Hydrogen storage cycling stability for MV(4.5)cnt(0.5) sample after five cycles of absorption (200 °C) and desorption (300 °C).

To elucidate this observation, dehydrogenation rate measurements of these MgH_2_-based composites were characterized at 300 °C and are shown in Figure 9. It clearly shows that a shorter CNTs milling time in the MV(4.5)cnt(0.5) sample improves the hydrogen desorption kinetics, for which 6.50 wt. % of hydrogen is released within 10 min in MV(4.5)cnt(0.5) sample as compared to only 6.00 wt. % hydrogen evolved in MVcnt5 sample (5 h CNTs milling time). It is also noteworthy that the presence of CNTs improves the hydrogen released capacity of about 7.00 wt. % compared to that of samples without addition of CNTs (6.50 wt. %), suggesting that CNTs allow more hydride available for dehydrogenation to take place. Moreover, Figure 10 shows the desorption rates of both samples based on the mass of the composites at a temperature range between 310 °C to 290 °C. It clearly illustrates that having a short 30 min CNTs milling time enhanced the desorption kinetics compared to a longer 5 h CNTs milling time at all desorption temperatures. Thus providing a direct relationship between defected CNTs structure availability and desorption performance. Complementing the observation made during absorption process the damaged CNTs structure contributes to the improvement in the rate of hydrogen desorption of the composites. Figure 11 shows that there are no difference of hydrogen storage property for MV(4.5)cnt(0.5) sample after five cycles of absorption (200 °C) and desorption (300 °C), indicating the excellent cycling property.

**Figure 12:**
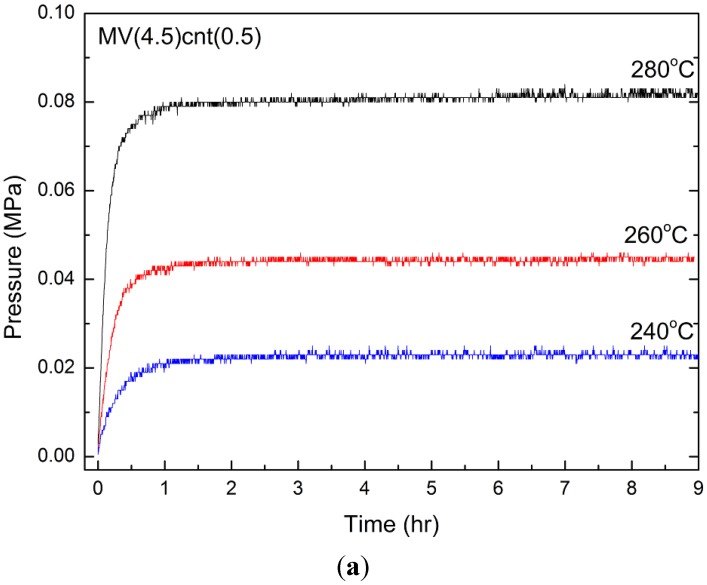

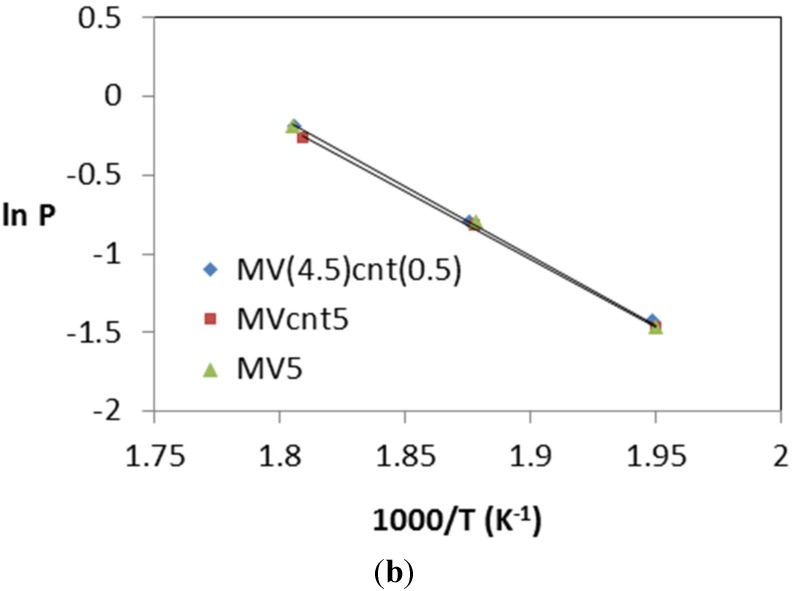
(**a**) Desorption kinetic plots of MV(4.5)cnt(0.5) showing that hydrogen desorption equilibrium was reached at all temperatures; (**b**) van’t Hoff plot for desorption of MV5, MVcnt5 and MV(4.5)cnt(0.5), respectively.

To further clarify the outstanding hydrogen desorption performance, the thermodynamic investigation was also carried out. A similar technique adopted from Paskevicius *et al.* [42] was used to identify the equilibrium desorption pressures at 280 °C, 260 °C and 240 °C for MV(4.5)cnt(0.5), MVcnt5 and MV5, respectively. Kinetic plots allow for an assessment of whether true equilibrium is met. The equilibrium desorption pressure curves of MV(4.5)cnt(0.5) are shown in Figure 12a. Since the equilibrium pressures for all samples are relatively low (less than 100 KPa), it is acceptable to assume that fugacity is equal to pressure. Thus a van’t Hoff plot was constructed using the equilibrium data obtained and are shown in Figure 12b.

The decomposition enthalpy (Δ*H*) and entropy (Δ*S*) are listed in Table 2. The effect of VCat towards thermodynamic stability can be observed by the reduction of both enthalpy and entropy of 73.9 kJ/mol H_2_ and 132.0 J/mol H_2_·K as compared to 78.5 kJ/mol H_2_ and 140.0 J/mol H_2_·K in the commercial MgH_2_ system, respectively [43]. As for co-milling MgH_2_ with CNTs, it was reported not to alter the enthalpy of desorption [23]. However, in this study it was found that adding VCat and CNTs to MgH_2_ reduces Δ*H* and Δ*S* further to at least 71.6 kJ/mol H_2_ and 127.4 J/mol H_2_·K, respectively, as shown for MVcnt5. This reduction of enthalpy is in accordance with the onset and peak temperature reduction observed in the TPD analysis (Figure 8). We are suggesting that thermodynamic properties are not affected by the CNTs milling time and the synergistic effect of VCat and CNTs are responsible for the improved thermodynamic properties. 

**Table 2:** Thermodynamic properties of various samples.

Sample	Desorption Enthalpy, Δ*H* (kJ/mol H_2_)	Desorption Entropy, Δ*S* (J/mol H_2_·K)
MV5	73.9	132.0
MVcnt5	71.6	127.4
MV(4.5)cnt(0.5)	72.2	128.8

## 4. Conclusions

In conclusion, we have successfully developed MgH_2_-based composite system incorporated with vanadium-based complex catalyst (VCat) and carbon nanotubes (CNTs) that exhibits improved hydrogen absorption/desorption properties with a shorter milling time of 5 h. From the study of the effect of CNTs milling time, it is shown that partially destroyed CNTs (shorter milling time) are likely to enhance the hydrogen sorption performance of the MgH_2_-based composites. The synergetic effect of VCat and CNTs can be explained by having both VCat as hydrogen splitting agent and the defected edge of CNTs as the active sites, whereas the tubular structure of the CNTs serves as hydrogen diffusion channels. An ultra-fast absorption rate of 6.50 wt. % of hydrogen is observed in 1 min at 200 °C, whereas 6.50 wt. % of hydrogen was released in 10 min at 300 °C in MV(4.5)cnt(0.5), based on the total mass of the MgH_2_-based composites. In addition, the presence of the VCat and CNTs reduces the enthalpy and entropy of desorption of about 7 kJ/mol H_2_ and 11 J/mol H_2_·K, respectively, as compared to the commercial MgH_2_. This may attribute to the improvement of hydrogen desorption of such a system.
